# Overcoming Gleevec-resistance by blocking oncogene addiction in malignant hematologic cells

**DOI:** 10.1186/1756-8722-5-S1-A6

**Published:** 2012-04-25

**Authors:** Jingxuan Pan

**Affiliations:** 1 Department of Pathophysiology, Zhongshan School of Medicine, Sun Yat-sen University, 74 Zhongshan Road II, Guangzhou 510089, People’s Republic of China

## 

In some types of tumors, malignant cells are highly dependent on the constitutive activation of a certain protein encoded by oncogene, despite existence of additional carcinogenic genetic changes. This phenomenon is referred to oncogene addiction. Typical examples include cytoplasmic tyrosine kinase Bcr-Abl in chronic myeloid leukemia (CML), receptor tyrosine kinase KIT in systemic mastocytosis and gastrointestinal stromal tumors (GISTs), and PDGFRα in hypereosinophilic syndrome (HES). In 2001, the approval of Gleevec (STI571, imatinib mesylate, Norvartis) by FDA (Food and Drug Administration, USA) initiated a revolutionary targeted therapy against cancer with small molecule tyrosine kinase inhibitors. Gleevec blocks the signaling pathway of tyrosine kinase by competitively occupying the ATP-binding pocket of Bcr-Abl, KIT and PDGFRα, and therefore kills these oncogene-addicted tumor cells. Patients with CML and HES have gained much better prognosis with the treatment of Gleevec. However, in some patients (particularly in accelerating and blast crisis phases), relapse due to resistance to Gleevec is an emerging problem.

Acquired point mutations within the target genes (Bcr-Abl, KIT and PDGFRα) are a major mechanism of resistance to Gleevec in some patients with hematologic malignance. The mutations are believed to block the binding of Gleevec to ATP binding pockets of these tyrosine kinases. In this case, novel tyrosine kinase inhibitor such as nilotinib and dasatinib (also called the second-generation of tyrosine kinase inhibitor) have been shown activity against Gleevec-resistant patients bearing some point mutations but the “gate-keeper” mutations (e.g., T315I Bcr-Abl, T674I PDGFRα). Therefore, development of more novel small molecule tyrosine kinase inhibitors is still needed.

This talk covered the advance in the field of overcoming Gleevec resistance in terms of novel compounds and strategeies. Pan J et al reported that EXEL-0862 is effective against Gleevec-resistant D816V KIT and T674I PDGFRα (2007). Recently, in vitro and animal data supported that several novel tyrosine kinase inhibitors including (but not limited) AP24534 (ponatinib) and DCC-2036 have been demonstrated effective against T315I Bcr-Abl. However, the efficacy and safety of these pounds (EXEL-0862, AP24534 and DCC-2036) in patients remains to be defined. An alternative approach for overcoming Gleevec-resistance is to decrease the expression of “addicted” oncogenes, which are driving forces of the tumor cells, to kill the malignant cells. Our group discovered several compounds which are effective against Gleevec-resistant tumor cells regardless of resistance to imatinib. The compounds kill cells harboring gate-keeper mutants of tyrosine kinases by lowering the expression of the oncoproteins (Bcr-Abl, KIT and PDGFRα). Examples include triptolide, pristimerin and SNS-032 (transcription inhibitors), homoharringtonine (a translation inhibitor), and celastrol (a hsp90 inhibitor).

In summary, Gleevec-resistance remains a challenge in leukemia. The findings from us and others suggest that several aforementioned compounds are promising agents to overcome Gleevec resistance, and warrant clinical trials.

